# Conventional twin studies overestimate the environmental differences between families relevant to educational attainment

**DOI:** 10.1038/s41539-023-00173-y

**Published:** 2023-07-17

**Authors:** Tobias Wolfram, Damien Morris

**Affiliations:** 1 Department of Sociology, University of Bielefeld, Niedersachen, Germany; 2 Department of Sociology, ENSAE/CREST, Paris, France; 3 Social, Genetic & Developmental Psychiatry Centre, King’s College London, London, United Kingdom

**Keywords:** Human behaviour, Education

## Abstract

Estimates of shared environmental influence on educational attainment (EA) using the Classical Twin Design (CTD) have been enlisted as genetically sensitive measures of unequal opportunity. However, key assumptions of the CTD appear violated for EA. In this study we compared CTD estimates of shared environmental influence on EA with estimates from a Nuclear Twin and Family Design (NTFD) in the same 982 German families. Our CTD model estimated shared environmental influence at 43%. After accounting for assortative mating, our best fitting NTFD model estimated shared environmental influence at 26%, disaggregating this into twin-specific shared environments (16%) and environmental influences shared by all siblings (10%). Only the sibling shared environment captures environmental influences that reliably differ between families, suggesting the CTD substantially overestimates between-family differences in educational opportunity. Moreover, parental education was found to have no environmental effect on offspring education once genetic influences were accounted for.

## Introduction

Educational attainment (i.e., ultimate years of education completed) is a key variable in the behavioural sciences because of its effectiveness in predicting a wide variety of important life outcomes. Despite being a measure that can be calculated from a single questionnaire item (e.g., “what is the highest qualification you’ve obtained?”) educational attainment (EA) is one of the best predictors of occupational status and income^1^, longevity and health outcomes^2^, and the risk of receiving a criminal conviction^3^. The qualities needed to advance through the modern secondary and tertiary education system appear to be useful for navigating a wide variety of challenges that life throws at individuals in advanced industrial economies.

One of the most established findings in the social sciences is that EA tends to run in families—a result which has widely been interpreted as evidence of persistent inequality in environmental opportunity and the “social reproduction” of socioeconomic advantages^4–8^. However, as noted by Jencks and Tach “the size of the correlation between the economic status of parents and their children is not a good indicator of how close a society has come to equalising opportunity… In particular, we must separate the contributions of genes” (p.2-3)^9^. From the 1970 s twin studies began to show evidence that the variation in EA had a substantial genetic component^10,11^. Two studies published in the last decade have sought to summarise the results of the international twin literature that has accumulated since then: a meta-analysis by Branigan et al.^12^ and a mega-analysis by Silventoinen et al.^13^ (see Supplementary Note 1). Both studies converged on similar results, estimating mean heritability at 40%–43% and mean shared environmental influence at 31%–36%. These heritability estimates are low relative to other highly correlated cognitive outcomes such as adult general cognitive ability (60%–80%)^14–16^ or adolescent school grades ( ~ 60% at age 16)^17^. However, the estimates of shared environmental influence are especially conspicuous, being among the highest for any behavioural trait investigated in adults.

That such high estimates have been reported for a socioeconomic outcome that bears on many important life chances has compelled some researchers to draw far-reaching conclusions about what this says about equality of opportunity in contemporary society. For example, after reporting high shared environmental estimates in their U.S. sample, Nielsen and Roos^18^ argued this “indicates a high level of inequality of opportunity for educational attainment in American Society at the turn of the twenty-first century” (p.535). However, a review paper by Freese and Jao^19^ cautioned against prematurely leaping to moralised conclusions about high estimates of shared environmental influence for EA when these might have innocuous explanations.

One possibility is that these are methodological artefacts. The mean international estimates of genetic and environmental influence on EA described above were calculated using variations on the Classical Twin Design (CTD). In CTD studies the variance in the target outcome is typically partitioned into additive genetic influence (*A*), shared environmental influence (*C*), and nonshared environmental influence (*E*) by comparing the resemblance of monozygotic (MZ) twins reared together with the resemblance of dizygotic (DZ) twins reared together. But just as estimates of the family environment’s influence on EA are confounded by unmodelled genetic influences in studies using parent-child or non-twin sibling correlations^12^, *ACE* estimates in the CTD are confounded by other unmodelled parameters that can potentially bias them up or down or affect their interpretation^20–22^. Two unmodelled parameters of particular interest in the present study are assortative mating and twin-specific shared environments.

One of the potential explanations for high *C* estimates of EA suggested by Freese & Jao (2017) was the presence of unmodelled assortative mating^19^. The CTD *ACE* model assumes random mating between spouses, attributing any additional resemblance shared by MZ twins relative to DZ twins to the additional 50% of their genes they are assumed to share [following Falconer’s formula *A* = 2(rMZ-rDZ)]^23^. Any residual resemblance between MZ twins after accounting for genetic influences is attributed to the shared environment (i.e. *C* = rMZ-*A*)^23^. However, under conditions of positive phenotypic assortment where spouses actively match on a heritable trait, this will induce a genetic correlation between spouses for that trait which also leads to higher genetic resemblance between their DZ twin offspring than the 50% kinship coefficient assumed under the CTD. This will cause the CTD to underestimate heritability and overestimate shared environmental influence.

EA exhibits some of the highest spousal correlations for any trait investigated, averaging r = 0.53^24^. However, phenotypic assortment is not the only possible explanation. Alternative explanations that do not imply increased genetic correlations between DZ twins are spousal convergence, in which partners become more similar over time due to their environmental influence upon each other; and social homogamy, in which the community from which individuals draw their partners resembles them for purely environmental reasons^25^. However, a large Australian study found spousal convergence played a negligible role in partner similarity for EA^26^, and recent molecular genetic studies have found strong evidence for phenotypic assortment on EA and associated traits^27–32^. A recent Norwegian study estimated the genetic correlation between spouses for EA at 0.37 and the genetic correlation between siblings at 0.67^31^—a value much larger than the expected correlation of 0.5, suggesting that CTD estimates of EA have been doubling the difference between MZ and DZ twin correlations to estimate heritability when *tripling* the difference might be more appropriate. Martin^33^ developed a method to correct CTD *ACE* estimates for bias due to phenotypic assortment when data on spousal correlations for parents is available. The authors of the Silventoinen et al.^13^ mega-analysis of 193,518 twins applied this adjustment to a subsample of 23,705 families with parent data (cross-parental correlations of 0.57). When they did so, the *C* estimate was driven to zero and all the *C* variance was re-allocated to the *A* estimate. The unadjusted *ACE* estimates for this subsample were not published in the paper but were almost identical with the full sample (*A* = 43%, *C* = 30%, and *E* = 27% vs. *A* = 43%, *C* = 31%, and *E* = 26%. Private correspondence with authors). To the extent that the spousal correlations for the wider sample are similar and phenotypic assortment explains that correlation, this potentially implies the mean *C* estimate in the main results for the mega-analysis should be entirely re-allocated to the *A* estimate, i.e.: *A* = 74%, *E* = 26%.

In Branigan et al.^12^, 13 of the 34 subgroups included in the meta-analysis were from studies that published spousal correlations for either the twins or their parents; however, the potential bias assortative mating introduced to *ACE* estimates in these studies was not explored. In Table 1 we recalculated the *ACE* estimates for each of these subgroups and adjusted them for assortative mating. We then replicated the fixed effects meta-analysis performed by Branigan et al.^12^ for this subsample, obtaining grand mean estimates for both the adjusted and the unadjusted *ACE* estimates (full details of this analysis are provided in Supplementary Note 2 and Supplementary Tables 1 and 8). The difference between our grand mean estimates in the adjusted vs. the unadjusted sample suggest, on average, *A* is biased downwards and *C* biased upwards by 16–17 percentage points in these CTD studies. Our grand mean *ACE* estimates for the unadjusted subsample are very similar to the headline results from the full sample in Branigan et al. (2013) suggesting the headline estimates may be biased to a similar extent (*A* = 38%, *C* = 39%, *E* = 22% in the subsample vs. *A* = 40%, *C* = 36%, *E* = 25% in the full sample).

**Table 1:** Evidence that assortative mating may be biasing estimates of genetic and shared environmental influence on educational attainment in the Branigan et al. (2013)^12^ meta-analysis.

Paper	Sex	Sample	Cohort	nMZ	nDZ	rMZ	rDZ	ACE estimates			rParents	Adjusted ACE estimates		
								A	C	E	µ	A	C	E
Heath et al. (1985)^72^^a^	Male	Norwegian Twin Panel	1915–1939	259	313	0.86	0.77	18%	68%	14%	0.86	22%	64%	14%
Heath et al. (1985)^72^^a^	Female	Norwegian Twin Panel	1915–1939	405	425	0.89	0.75	28%	61%	11%	0.86	47%	42%	11%
Heath et al. (1985)^72^^a^	Male	Norwegian Twin Panel	1940–1949	253	284	0.82	0.48	68%	14%	18%	0.72	82%	0%	18%
Heath et al. (1985)^72^^a^	Female	Norwegian Twin Panel	1940–1949	342	400	0.85	0.68	34%	51%	15%	0.72	59%	26%	15%
Heath et al. (1985)^72^^a^	Male	Norwegian Twin Panel	1950–1960	370	463	0.85	0.47	76%	9%	15%	0.73	85%	0%	15%
Heath et al. (1985)^72^^a^	Female	Norwegian Twin Panel	1950–1960	518	576	0.89	0.66	46%	43%	11%	0.73	89%	0%	11%
Lykken et al. (1990)^73^	Male	Minnesota Twin Registry	1936–1955	433	632	0.64	0.44	40%	24%	36%	0.55	59%	5%	36%
Lykken et al. (1990)^73^	Female	Minnesota Twin Registry	1936–1955	392	571	0.66	0.5	32%	34%	34%	0.55	41%	25%	34%
Baker et al. (1996)^74^^b,c^	Male	Australian Twin Register	1893–1950	216	94	0.7	0.53	34%	36%	30%	0.426	41%	29%	30%
Baker et al. (1996)^74^^b,c^	Female	Australian Twin Register	1893–1950	520	299	0.77	0.55	44%	33%	23%	0.426	59%	18%	23%
Baker et al. (1996)^74^^b,c^	Male	Australian Twin Register	1951–1965	226	161	0.74	0.47	54%	20%	26%	0.426	74%	0%	26%
Baker et al. (1996)^74^^b,c^	Female	Australian Twin Register	1951–1965	479	290	0.75	0.49	52%	23%	25%	0.426	75%	0%	25%
Bingley et al. (2005)^75^^c^	Male	Danish Twins Registry	1925–1977	2185	3534	0.62	0.444	35%	27%	38%	0.392	42%	20%	38%
**Grand mean**								**38%**	**39%**	**22%**		**55%**	**23%**	**22%**

*nMZ* number of monozygotic twin pairs, *nDZ* number of dizygotic twin pairs, *rMZ* correlation between MZ twins, *rDZ* correlation between DZ twins, *rParents* correlation between parents. *A* = additive genetic influence, *C* = shared environmental influence, *E* = nonshared environmental influence.

^a^Correlations in Heath et al. (1985)^72^ were polychoric. As no distinction was made between *ACE* estimates derived from Pearson correlations and polychoric correlations in Branigan et al. (2013)^12^ we treat both correlations interchangeably in this re-analysis. We use the µ path estimate from the model-fitting results in Heath et al. (1985)^25^ which corrects rParents for biased reports of parental education from each twin.

^b^Spousal correlations were only published for the full sample for Baker et al. (1996)^74^ but we assume here that they were the same for each gender and birth cohort.

^c^Correlations between twins and spouses were used for rParents in Baker et al. (1996)^74^ and Bingley et al. (2005)^75^ because correlations between parents were unavailable.

This table presents data from a subsample of studies included in Branigan et al.^12^ that reported correlations between parents or spouses for educational attainment. For each entry, we calculated *ACE* estimates using Falconer’s formulas^23^ then adjusted these estimates for assortative mating using a formula from Martin^33^. We then replicated the Branigan et al.^12^ meta-analysis for both the adjusted and unadjusted *ACE* estimates. After adjusting for assortative mating, mean *A* estimates were 17 points higher and mean *C* estimates were 16 points lower. Grand mean *ACE* estimates are shown in bold. Full details on the methodology are provided in Supplementary Note 2 and full workings are provided in Supplementary Table 1.

Twin-specific shared environmental influence is another unmodelled parameter in CTD studies that has important implications for how CTD estimates of shared environmental influence are interpreted. In this study, twin-specific shared environments refers to environmental influences held in common by twins which are experienced as nonshared environmental influences by siblings growing up at different times. These will include the effects of, e.g.: birth order relative to other siblings; “birthday effects” of being born earlier or later in the year; and the cohort effects of being born in a particular political, economic, or cultural epoch. Kendler et al. (2019)^34^ invoked twin-specific shared environments alongside assortative mating as a potential explanation for why CTD estimates of *C* were 11–12 percentage points higher for EA than estimates from half- or step-sibling study designs using the same Swedish register data.

A longstanding convention in CTD studies is to interpret *C* estimates as a measure of “between-family” environments which “make members of a family…similar to one another and different from members of other families” (p.18)^35^. For EA, this convention leads to *C* being interpreted as a measure of inequality of environmental opportunity *between* families, e.g., Nielsen and Roos (2015)^18^ write: “The shared environment component … has a direct policy interpretation: it reflects the potential effect on educational attainment of raising the quality of the most disadvantaged family environments to the level of the most advantaged ones” (p.539). But to the extent that *C* captures twin-specific shared environments, it will also capture environmental effects that make siblings in the *same* family different from each other, making them an inflated estimate of between-family environmental differences. Moreover, while twin-specific shared environments will capture real inequalities of opportunity between siblings, these within-family differences in opportunity are not the kind that ordinarily preoccupy policymakers or advocacy groups, who tend to be more concerned about between-family differences in, e.g., parental income, education, or occupational status^36^.

The presence of twin-specific shared environments (*T*) can be detected by incorporating data from DZ twins and their non-twin siblings in the same study, with *T* indicated when DZ twins resemble each other more closely than non-twin siblings. These effects have previously been reported for a US twin and sibling study of EA^18^ which found that *C* was 11.3% higher (and *E c*orrespondingly 11.3% lower) for twins than for non-twin siblings when accounting for *T*. The supplements of the first Genome-Wide Association Study (GWAS) of EA also included a twin and sibling analysis of the Swedish Multigenerational Registry, which reported that *T* accounted for 6.2% of the variance^37^. Furthermore, when we compared sibling correlations for EA from a recent international study^8^ with DZ twin correlations from studies in the same countries with similar birth cohorts, the DZ twin correlations were invariably higher, suggesting twin-specific environments might be a general phenomenon for this outcome (see Table 2).

**Table 2:** Correlations for educational attainment are consistently higher between dizygotic twins than between non-twin siblings suggesting twin-specific shared environments influence this trait.

Sibling studies				Twin studies			
Paper	Country (Birth year)	Sample	rSIB	rDZ	Country (Birth year)	Sample	Paper
Gratz et al. (2021)^8^	Finland (1974–1980)	Registers (Statistics Finland)	0.36	0.52^a^	Finland (1974–1979)	Finn16	Silventoinen et al. (2020)^13^
				0.60^b^	Finland (1936–1955)	Finnish Twin Cohort Study	Silventoinen et al. (2004)^76^
				0.62^b^	Finland (1919–1957)	Finnish Twin Cohort Study	Silventoinen et al. (2000)^77^
	Germany (1976–1989)	SOEP	0.51	0.60^a^	Germany (1990–1993)	TwinLife	Baier & Lang (2019)^60^
				0.60^a^	Germany (1914–1969)	Bielefeld Longitudinal Study	Silventoinen et al. (2020)^13^
				0.67^a^	Germany (1926–1987)	Berlin Twin Register	
	Norway (1970–1980)	Registers	0.41	0.51^b^	Norway (1967–1979)	Norwegian Twin Register	Ørstavik et al. (2014)^78^
				0.46	Norway (1967–1979)	Norwegian Twin Register	Lyngstad et al. (2017)^79^
	Sweden (1960–1982)	Multi-generation Registry	0.44	0.55	Sweden (1926–1958)	Swedish Twin Registry	Isacsson (1999)^80^
				0.50^c^	Sweden (1950–1970)	Multi-generation Registry	Rietveld et al. (2013)^37^
	UK (1954–1989)	UKHLS	0.42	0.47	UK (1951–1985)	TwinsUK	Branigan et al. (2013)^12^
	USA (1954–1986)	PSID	0.51	0.56	USA (1976–1984)	AddHealth	Nielsen & Roos (2015)^18^
				0.52^a^	USA (1908–1977)	California Twin Programme	Silventoinen et al. (2020)^13^
				0.54^a^	USA (1910–1976)	Carolina African American	
				0.56^a^	USA (1979–1989)	Colorado Twin Registry	
				0.61^a^	USA (1894–1987)	Mid Atlantic Twin Registry	

			Twin and sibling studies				
			rSIB	rDZ	Country (Birth year)	Sample	Paper
			0.39	0.49	Norway (1946-65)	Registers	Björklund & Salvanes (2011)^61^
			0.45	0.50	Sweden (1950–1970)	Multi-generation Registry	Rietveld et al. (2013)^37^
			0.45	0.56	USA (1976–1984)	AddHealth	Nielsen & Roos (2015)^18^

rSIB represents the Pearson correlation for years of education between siblings and rDZ for the correlation between dizygotic twins.

^a^rDZ was algebraically derived from *ACE* estimates using the formula 0.5 *A* + *C*.

^b^rDZ was pooled from male and female samples (simple arithmetic mean and inverse variance weighted averages were the same).

^c^rDZ is male only (while rSIB is mixed).

In this study, we used a Nuclear Twin and Family Design (NTFD) to account for both assortative mating and twin-specific shared environments using data on twins, their parents, and their siblings in the German TwinLife sample. Moreover, unlike the CTD, NTFD models can estimate non-additive genetic influences (*N*) and shared environmental influences simultaneously. Unmodelled *N* can bias estimates of heritability upwards in the CTD *ACE* model and bias estimates of shared environmental influence downwards. The degree of bias introduced depends on whether these unmodelled non-additive genetic influences consist of gene-gene interactions at single loci (“dominance”) or interactions across multiple loci (“epistasis”).

Furthermore, NTFD models can disaggregate phenotypic transmission (*P*)—here the environmental effects of parental education on offspring education—from other twin or sibling shared environments. NTFD models can likewise disaggregate the variance explained by passive gene-environment correlation (rGE), which is captured under the *C*-component in the CTD *ACE* model. The contribution of passive rGE to EA is a subject of growing scientific interest as molecular genetic studies have indicated it might explain around half of the phenotypic variation captured by current EA polygenic scores (PGSs)^29,38–41^. We compare the results from NTFD and CTD models run on EA data from the same families in order to assess the size and direction of bias in our CTD parameter estimates.

A previous TwinLife study by Eifler and Riemann (2021)^42^ used an NTFD phenotypic assortment model to decompose the variance in school leaving certificates. Here we extend that work to decompose ultimate years of education completed as imputed from both completed qualifications and enrolled post-secondary education courses. We further build on that analysis by contrasting NTFD results with CTD results, by fitting social homogamy models in addition to phenotypic assortment models, and by modelling both dominance and epistasis as potential sources of non-additive genetic influence. By exploring a wider range of boundary conditions in which different assumptions are made and different parameters are estimated, we have attempted to map out the plausible parameter space defined by NTFD models of these data^22^.

## Results

### Correlations between different relatives

Correlations for EA between different relatives in our sample are presented in Table 3. MZ twins were highly correlated (r = 0.77) suggesting substantial familial (i.e., genetic and/or environmental) influences on the trait. DZ twins were somewhat less correlated than MZ twins (r = 0.6) suggesting that some of the familial influence is genetic, but most is due to shared environmental influence. However, mothers and fathers were also highly correlated with each other (r = 0.6), suggesting that assortative mating of some kind is present. This could imply that genetic influence is higher, and shared environmental influence lower, than CTD *ACE* estimates would normally imply. Additionally, DZ twin correlations (r = 0.6) were substantially higher than the correlations between twins and their non-twin siblings (r = 0.45), suggesting that twin-specific shared environmental influences might play an important role.

**Table 3:** Correlations for educational attainment between different types of family members.

Dyads	r (95% CIs)	Number of pairs
MZ twins	0.769 (0.73–0.803)	498
DZ twins	0.595 (0.531–0.652)	439
Sibling and random twin	0.453 (0.339–0.554)	212
Mother and random twin	0.406 (0.35–0.46)	884
Mother and sibling	0.497 (0.385–0.594)	201
Father and random twin	0.418 (0.345–0.486)	525
Father and sibling	0.388 (0.232–0.524)	132
Parents	0.597 (0.536–0.651)	482

r = Pearson correlation, *CIs* Confidence Intervals. The *p* value was <0.001 for all correlations.

### Model fitting results

Based on the twin correlations above, in which the MZ twin correlations were less than twice as large as the DZ twin or sibling correlations, we proceeded to fit a CTD *ACE* model to our twin only data rather than an *ANE* model that estimates non-additive genetic influences instead of shared environmental influences. This produced estimates of: *A* = 34% (95% CIs: 23–47%), *C* = 43% (31–53%), and *E* = 23% (20–26%).

We then fit NTFD models to our full twin, parent, and sibling data. Three phenotypic assortment (PA) models and three social homogamy (SH) models were compared against a saturated model, respectively fixing non-additive genetic effects (*N*), sibling shared environments (S), and phenotypic transmission (*P*) to zero, as only two of these three parameters can be estimated simultaneously^20^. None of these six baseline models fit the data significantly worse than the saturated model. We proceeded to drop all non-significant paths from each of the baseline models to see if doing so produced a significant reduction in fit. It did not. Model fitting results are presented in Table 4.

**Table 4:** Fit indices for Nuclear Twin and Family Design models.

Ref #	Model name	Fixed parameters	-2LL	df	AIC	BIC	Against	Δ df	Δ -2LL	*p*-value
Full	Saturated		8,981.36	0	9,064.84	9,129.90				
PA-1	ASTPE	N = 0	9,006.71	3570	9028.98	9047.56	Full	39	25.35	0.96
PA-1.1	ASTPE (no rGE)	N = rGE = 0	9,006.71	3571	9026.93	9043.84	PA-1	1	0.00	0.96
**PA-1.2**	**ASTE**	**N** = **P** = **0**	**9,006.71**	**3573**	**9022.86**	**9036.42**	**PA-1**	**3**	**0.00**	**1.00**
PA-2	ANTPE	S = 0	9,010.47	3570	9032.75	9051.32	Full	39	29.12	0.88
PA-2.1	ANTPE (no rGE)	S = rGE = 0	9,010.56	3571	9030.79	9047.70	PA-2	1	0.09	0.77
PA-2.2	ATPE	S = N = 0	9,010.63	3571	9030.86	9047.77	PA-2	1	0.16	0.69
PA-2.3	ATPE (no rGE)	S = N = rGE = 0	9,010.63	3572	9028.82	9044.05	PA-2	2	0.16	0.92
PA-2.4	ANTE	S = P = 0	9,010.56	3573	9026.71	9040.27	PA-2	3	0.09	0.99
PA-2.5	ATE	S = N = P = 0	9,010.63	3574	9024.75	9036.63	PA-2	4	0.16	1.00
PA-3	ANSTE	P = 0	9,006.71	3570	9028.98	9047.56	Full	39	25.35	0.96
**PA-3.1**	**ASTE**	**P** = **N** = **0**	**9,006.71**	**3573**	**9022.86**	**9036.42**	**PA-3**	**3**	**0.00**	**1.00**
*SH-1*	*ASTPE*	*N* = *0*	*9,006.71*	*3570*	*9028.98*	*9047.56*	*Full*	*39*	*25.35*	*0.96*
SH-2	ANTPE	S = 0	9,011.19	3570	9033.46	9052.03	Full	39	29.83	0.85
SH-2.1	ATPE	S = N = 0	9,011.19	3571	9031.41	9048.32	SH-2	1	0.00	1.00
SH-3	ANSTE	P = 0	9,034.37	3570	9056.64	9075.21	Full	39	53.01	0.07
SH-3.1	ASTE	P = N = 0	9,034.36	3573	9050.51	9064.07	SH-3	3	0.00	1.00
SH-3.2	ANSE	P = T = 0	9,034.95	3573	9051.10	9064.66	SH-3	3	0.58	0.90
SH-3.3	ASE	P = N = T = 0	9,034.95	3574	9049.06	9060.94	SH-3	4	0.58	0.96

*PA* Phenotypic Assortment model, *SH* Social Homogamy model, *A* Additive genetic influence, *N* non-additive genetic influence (dominance), *S* environmental influences shared by all siblings, *T* environmental influences shared by twins only, *P* phenotypic transmission, *E* nonshared environment, *rGE* passive gene-environment correlation, *-2LL* negative 2 log likelihood, *df* degrees of freedom, *AIC* Akaike’s Information Criterion, *BIC* Bayesian Information Criterion, Δ difference.

Non-additive genetic influences, sibling shared environmental influences, and phenotypic transmission effects were respectively set to zero in three baseline Phenotypic Assortment models and three baseline Social Homogamy models (top row of each section). These were compared with a saturated model (top row). For baseline models which did not show a significant reduction in fit (α = 0.05), non-significant paths were iteratively dropped to see if nested sub-models showed a significant reduction in fit relative to baseline. Our best-fitting model (*ASTE*) is in boldface and appears twice under PA-1.2 and PA-3.1. Our best-fitting SH model (*ASTPE*) is in italics. Alternative model-fitting results where non-additive genetic influences were characterised as epistasis rather than dominance are provided in Supplementary Table 3, but differences were negligible. For our P = 0 baseline models only one path is dropped (m) but for all P = 0 submodels three paths have been dropped (m, x, and w). This is because the value of x and w automatically fall to zero when m is dropped (see path diagram in Fig. 3) and parameter estimates remained the same whether one, two, or three paths were dropped. We have omitted redundant P = 0 submodels that were less parsimonious.

Our PA models returned mean estimates of additive genetic influence ranging between 51–56%, non-additive genetic influences of 0–1%, parental influence of 0–1%, passive rGE of −2%−0%, sibling shared environments of 0–10%, twin-specific shared environments of 16–25%, and nonshared environments of 23%. Non-additive genetic influences, parental influences, and passive rGE could be dropped from all three PA baseline models without producing a significant decline in fit.

Our SH models returned mean estimates of additive genetic influence of 36–70%, zero non-additive genetic influence, phenotypic transmission of 0–4%, passive rGE of 0–10%, sibling shared environments of 0–11%, twin-specific shared environments of 0–25%, and nonshared environments of 21–23%. Phenotypic transmission was statistically significant in the two SH baseline models in which it was freely estimated and was therefore retained in the corresponding submodels. The SH baseline model that fixed phenotypic transmission to zero was our worst-fitting model and also yielded unusual results, e.g., producing heritability estimates even higher than our PA models (69%). Without this model or its nested submodels the SH heritability estimates range from 36–39%, substantially lower than our PA estimates and closer to our CTD estimate of 34%.

Under all PA models the parent-offspring correlation for EA was entirely genetically mediated. Under our SH models 43–46% of the parent-offspring correlation was genetically mediated except in our worst-fitting models where phenotypic transmission was fixed to zero (see Supplementary Table 4).

In general, SH models fit the data slightly worse than our PA models; however, our SH baseline model which assumed no non-additive genetic influence fit the data marginally better than PA models which assumed no sibling effects (see AIC and BIC values in Table 4). Our best fitting model overall, reporting both the lowest Akaike’s Information Criterion and lowest Bayesian Information Criterion^43,44^, was the PA *ASTE* model where *A* = 51% (46–56%), *S* = 10% (0.1–18%), *T* = 16% (8–26%), and *E* = 23% (21–26%).

**Fig. 1:**
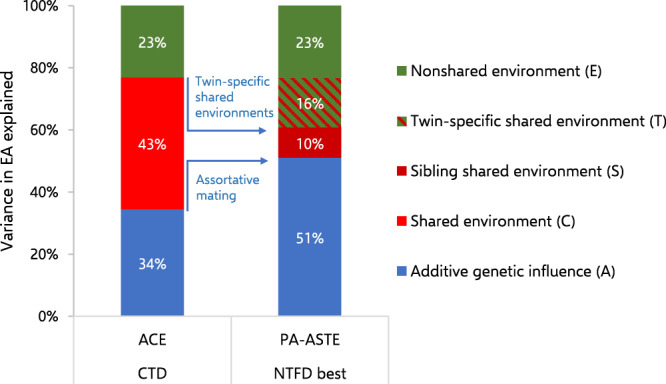
Comparing estimates of genetic and environmental influence for educational attainment (EA) in the Classical Twin Design (CTD) with estimates from a Nuclear Twin and Family Design (NTFD). Our best fitting NTFD model showed higher additive genetic influence and lower shared environmental influence compared to the CTD model after adjusting for phenotypic assortment (PA). In addition, our best fitting NTFD model found that a substantial fraction of the variance attributed to shared environmental influence in the CTD model consisted of twin-specific shared environments that non-twin siblings do not hold in common, and which cannot be safely interpreted as “between-family” environmental differences.

**Fig. 2:**
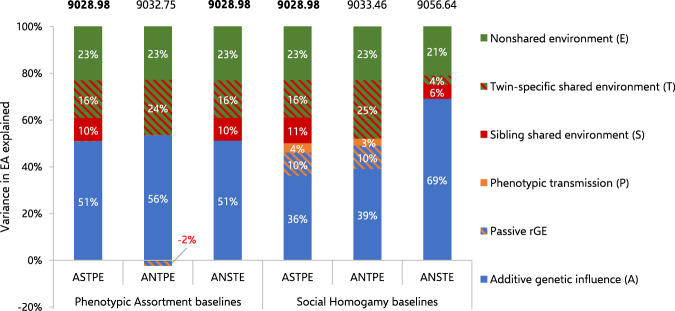
Comparing estimates of genetic and environmental influence for educational attainment (EA) across different baseline models in the Nuclear Twin and Family Design (NTFD). Here we depict our three Phenotypic Assortment (PA) and three Social Homogamy (SH) baseline models in which alternative parameters were fixed to zero for model identification purposes. Akaike’s Information Criteria (AIC) values are displayed above each column. Three baseline models were tied for the best fit (AIC values provided in bold). The variance components in our two best fitting PA baseline models were almost identical with the variance components in our best fitting model overall (see Fig. 1). These estimates can be compared against the variance components in our best fitting SH model (the SH-*ASTPE* baseline) in which ~14% of the variance shifts from additive genetic influence to phenotypic transmission and passive gene-environment correlation (rGE) while other estimates remain broadly the same. In the PA-*ANTPE* model, additive genetic influence should be interpreted as overlapping with negative rGE, which reverses its effects.

Our best fitting NTFD model is contrasted against the CTD *ACE* model in Fig. 1. Our six NTFD baseline models are compared in Fig. 2. Variance components and confidence intervals for all NTFD models are presented in Table 5. Finally, the path estimates (with standard errors) for all NTFD models are available in Supplementary Table 2.

**Table 5:** Variance components for Educational Attainment in our Nuclear Twin and Family Design models.

Ref #	Model name	A	N	S	T	P	rGE	E
PA-1	ASTPE	51% (32–56%)		10% (0–19%)	16% (8–27%)	0% (0–2%)	0% (0–9%)	23% (21–26%)
PA-1.1	ASTPE (no rGE)	51% (46–56%)		10% (0–18%)	16% (8–26%)	0% (0–0%)		23% (21–26%)
**PA-1.2**	**ASTE**	**51% (46-56%)**		**10% (0–18%)**	**16% (8–26%)**			**23% (21–26%)**
PA-2	ANTPE	56% (8–56%)	0% (0-0%)		24% (16–32%)	0% (0–13%)	−2% (NA–12%)	23% (21–26%)
PA-2.1	ANTPE (no rGE)	52% (47–56%)	1% (0–10%)		24% (16–30%)	0% (0-0%)		23% (20–26%)
PA-2.2	ATPE	52% (37–60%)			25% (20–32%)	0% (0–0%)	0% (−1–8%)	23% (21–26%)
PA-2.3	ATPE (no rGE)	52% (47–56%)			25% (20–30%)	0% (0-0%)		23% (21–26%)
PA-2.4	ANTE	52% (47–56%)	0% (0–10%)		24% (16–30%)			23% (21–26%)
PA-2.5	ATE	52% (47–56%)			25% (20–30%)			23% (21–26%)
PA-3	ANSTE	51% (46–56%)	0% (0–8%)	10% (0–18%)	16% (7–26%)			23% (21–26%)
**PA-3.1**	**ASTE**	**51% (46–56%)**		**10% (0–18%)**	**16% (8–26%)**			**23% (21–26%)**
*SH-1*	*ASTPE*	*36% (25–49%)*		*11% (1–19%)*	*16% (7–27%)*	*4% (1–7%)*	*10% (7–12%)*	*23% (21–26%)*
SH-2	ANTPE	39% (5–52%)	0% (0–0%)		25% (17–32%)	3% (2–17%)	10% (7–12%)	23% (20–26%)
SH-2.1	ATPE	39% (28–52%)			25% (17–32%)	3% (1–7%)	10% (7–12%)	23% (20–26%)
SH-3	ANSTE	69% (63–76%)	0% (0–2%)	6% (0–15%)	4% (0–14%)			21% (19–24%)
SH-3.1	ASTE	69% (61–76%)		6% (0–15%)	4% (0–14%)			21% (19–24%)
SH-3.2	ANSE	70% (63–76%)	0% (0–3%)	9% (2–16%)				22% (19–24%)
SH-3.3	ASE	70% (63–76%)		9% (2–16%)				22% (19–24%)

*PA* phenotypic assortment model, *SH* social homogamy model, *A* additive genetic influence, *N* non-additive genetic influence (dominance), *S* environmental influences shared by all siblings, *T* environment shared by twins only, *P* effects of parental education, *rGE* passive gene-environment correlation, *E* nonshared environment.

Results for both Phenotypic Assortment (PA) models and Social Homogamy (SH) models are provided. Baseline models in which N, S, or P have been fixed to zero for model-identification purposes appear in the top row of each section. Our overall best fitting model (PA-*ASTE*) is in boldface and appears twice under PA-1.2 and PA-3.1. Our best fitting SH model (SH-*ASTPE*) is italicised. 95% confidence intervals are in parentheses. Alternative results where non-additive genetic influences were characterised as epistasis are provided in Supplementary Table 3, but differences were negligible.

In addition to the results displayed here which assume that non-additive genetic influence is characterised by dominance we also ran an alternative set of epistatic models which assumed it was characterised by multi-local gene–gene interactions that only MZ twins share in common (see Supplementary Tables 3 and 5). This scenario isn’t considered biologically plausible but ensures that non-additive genetic effects shared by DZ twins aren’t over-estimated^22^. There were negligible differences between the dominance and epistatic model results.

## Discussion

We set out to explore how NTFD estimates of genetic and environmental influence for EA differed from conventional CTD estimates when the inclusion of more relative classes allowed additional parameters to be estimated. When phenotypic assortment (PA) was assumed, broad heritability estimates ranged from 51% to 56% in our NTFD models. Our best-fitting model estimated heritability at 51%, up 17 percentage points from our CTD estimate of 34%. This difference aligns with the 17-point assortative mating adjustment to heritability that we calculated in our re-analysis of studies in Branigan et al.^12^ (see Table 1). Together these results indicate that the 40% and 43% mean heritability estimates for EA reported in Branigan et al.^12^ and Silventoinen et al.^13^ might underestimate the true international average heritability for the relevant populations.

If the mean heritability of EA is ~17 percentage points higher than previously believed, this could also indicate that the ceiling on polygenic prediction for EA is higher than previously assumed. While the variance explained by PGSs (12–16% depending on cohort)^29^ is already approaching the current SNP heritability for EA (averaging ~15% globally)^45^, as whole genome sequencing of large samples becomes widespread, and rarer variants associated with EA are identified, it’s expected that both the SNP heritability and the variance explained by future EA PGSs will increase^46–48^. Pedigree-based estimates of heritability therefore provide an optimistic upper bound for the strength of the polygenic prediction that might ultimately be achieved.

Our NTFD results also suggest that CTD estimates of shared environmental influence (*C*) for EA might be overestimated. Total shared environmental influence (including passive rGE) was 26% in our best-fitting model, down 17 points from our CTD estimate of 43% after accounting for phenotypic assortment. Again, this aligns closely with our assortative mating adjustment to the Branigan et al. (2013) *ACE* estimates. Once we consider the growing evidence for genetic correlations between spouses for EA^27–32^ and the high spousal correlations for the studies included in Branigan et al. (2013)^12^ and Silventoinen et al. (2020)^13^, it suggests the 31–36% mean international estimates of shared environmental influence for EA in those studies might be substantially inflated.

However, our results also indicate that CTD estimates of shared environmental influence on EA cannot be safely interpreted as “between-family” differences in environmental opportunity irrespective of whether unmodelled assortative mating is an issue. Our NTFD models were able to decompose the shared environment into variance components that are shared by non-twin siblings (i.e., *S*, *P*, and rGE) and twin-specific shared environments (*T*) that are not. *T* accounted for 16–25% of the variance in our PA models and contributed a similar range in our SH models (when our worst fitting SH models that set parental effects to zero were excluded). Taking both assortative mating and twin-specific shared environments into account, our best-fitting model indicated just 10% of the variance in EA could be attributed to between-family environmental differences. This is 33 points lower than our *C* estimate of 43% under the CTD. Our survey of DZ twin versus non-twin sibling correlations for EA in Table 2 indicates that twin-specific shared environments are relevant for many of the populations in which CTD studies of EA have previously been conducted. These results suggest that researchers should refrain from drawing strong conclusions about the differences in educational opportunity between families based on CTD estimates for EA^12,13,49^.

Additionally, the decomposition of the shared environment under our NTFD PA models (which include our best-fitting model) implied negligible environmental influence of parental education on offspring education. Under these models, the observed parent-offspring correlation was entirely genetically mediated (see Supplementary Table 4) inverting the traditional sociological interpretation that this correlation captures environmental inequalities^4–8^. However, this does not imply that parents have no effect on offspring EA. Parental attributes other than EA could be driving some of the phenotypic similarity between siblings and between twins that is captured under *S* and *T* and those parental attributes could potentially include alternative socioeconomic indicators such as parental income.

Full genetic mediation of the parent-offspring correlation for EA was also found in a recent Norwegian study using a Multiple Children of Twins design^50^. However that study speculated that this was the result of Norway’s egalitarian social policies and specifically predicted that the more stratified German education system would produce different results^50^. Instead, our results indicate that genetic mediation of the parent-offspring correlation might be a more general phenomenon. That would suggest the intergenerational mobility literature exaggerates the environmental transmission of advantage and the differences in opportunity between families even more than CTD studies have previously indicated^12^.

For over 60 years it has been common practice in the social sciences to treat the correlation for EA between first-degree relatives as a direct measure of inequality of environmental opportunity, painting a picture of society that is deeply and persistently unmeritocratic^4–8^. By demonstrating that a substantial fraction of the familial correlation is genetic, CTD studies have shown that environmental differences between families play a much smaller role in the intergenerational persistence of EA than has sometimes been suggested^12^. Nevertheless, conspicuously high CTD estimates of shared environmental influence for EA have continued to cause concern about high levels of unequal opportunity for this outcome^13,18,51^. The results presented here suggest that shared environmental influence might account for even less of the variation in educational attainment than conventional twin studies have indicated and that environmental opportunities might therefore be more equal than these studies have implied. Moreover, a large fraction of the remaining shared environmental variation for EA appears to consist of twin-specific shared environments that capture within-family differences in opportunity that carry a different moral and political connotation to between-family differences (even if they remain potential targets for political intervention). A promising avenue for future research would be to identify specific environmental variables which account for these within- and between-family differences in educational opportunity^52,53^.

That noted, we stress that equality of environmental opportunity—while a widely endorsed social goal—is not an uncontested one. Some have argued for a more radical egalitarian agenda that seeks to reduce the influence of both environmental and *genetic* accidents of birth on socially valued outcomes^54–56^. Others have argued that promoting conditions that maximise general welfare and personal freedom should take precedence over attempts to reduce environmental differences between people^57–59^. These important philosophical debates are, however, beyond the scope of this paper.

Our study involved the following limitations. By assuming subjects who are enroled in ongoing post-secondary studies go on to complete those courses, we potentially introduce bias by failing to capture dropouts. However, if we make stricter assumptions and only use the level of education completed, this severely reduces the variance in years of education (because of the youth of our sample). This is also an unrealistic assumption about the educational trajectory of subjects enrolled in post-secondary education given low German drop-out rates and a tendency for students and trainees to transfer horizontally into an alternative vocational or tertiary qualification rather than making a vertical change between categories^60^. Follow-up studies when the cohort is older will be able to address this limitation.

In addition, the negligible effect of parental EA on offspring EA under our PA models contradicts the evidence from studies which find a significant association between the EA of adoptive parents and adoptive children^61,62^. Here we stress that, while our best-fitting model was a PA model, our SH models also fit the data. It’s possible that a mixed homogamy scenario, in which phenotypic assortment and social homogamy both play a role, might explain the data better than the PA and SH models compared in this study. If so, that would suggest that the true contribution of genetic and environmental influences to the parent-offspring correlation and to the variance in EA lies somewhere between the PA and SH estimates presented here. This might also explain why our best-fitting model indicates no passive rGE in contrast to molecular genetic literature that suggests that EA polygenic scores partly capture passive rGE^28,29,38–41^; however, we also note that phenotypic assortment is expected to produce some of the molecular genetic effects that have been interpreted as passive rGE or “genetic nurture”^28,38,63^.

We also stress that the biases in CTD parameter estimates that we have reported for EA will not necessarily generalise to other traits. The size and direction of these biases can vary considerably across different traits depending on the extent to which different assumptions in the CTD model are violated.

In summary, by comparing the estimates of genetic and environmental influence on Educational Attainment (EA) from a Nuclear Twin and Family Design with the results from a conventional twin-only study in the same German families, we were able to account for some potential confounds in the Classical Twin Design (CTD). Our results indicate that unmodelled assortative mating may be introducing substantial downwards bias into CTD estimates of heritability for EA while correspondingly biasing estimates of shared environmental influence upwards. Our results also indicate that twin-specific shared environments might account for a substantial portion of the shared environmental estimate in CTD studies of EA, suggesting that such estimates cannot be safely interpreted as between-family differences in environmental opportunity. Our survey of previous CTD studies of EA suggest both issues are likely to generalise beyond our TwinLife sample, as we find high spousal correlations in those studies and high DZ twin correlations relative to non-twin sibling correlations in comparable samples. Together these findings suggest the differences in educational opportunity between families are substantially lower than CTD estimates of shared environmental influence on EA have indicated. In addition, we found that the relatively high parent-offspring correlation for EA in our German sample was fully explained by genetic transmission under our best fitting model, suggesting parental education might not be the engine of social reproduction of advantage that many sociological studies have implied.

## Methods

### Sample

All analyses were performed on data from TwinLife: a cross-sequential panel-study of German twins and their immediate relatives (parents, spouses, and the nearest sibling by age). TwinLife is broadly representative of twin and multiple-birth households in Germany^64^. The full sample consists of 4,097 twin pairs spanning four birth cohorts (born 1990–1993, 1997–1998, 2003–2004 and 2009–2010). Since its inception in 2014, data on participating twins and their relatives has been collected every year with face-to-face interviews and telephone interviews taking place on an alternating biennial basis. For this study, we used data from the oldest 1990–1993 cohort of twins (and relatives) only. We only used data on siblings who were born less than five apart from the twins in any given family to ensure our results were not primarily driven by outliers with large sibship-age differences. Data on educational attainment was available for 1,020 MZ twins (498 complete pairs), 896 DZ twins (439 complete pairs), 215 siblings, 906 mothers, and 536 fathers. Descriptive statistics are provided in Table 6.

**Table 6:** Descriptive statistics for our TwinLife sample.

	Total	Female	Male	Pairs	Age	Mean EA	SD of EA
MZ twins	1020	610	410	498	22–27	14.7	2.94
DZ twins	896	511	385	439	22–27	14.4	3.05
Siblings	215	116	99	0	21–30	14.8	3.09
Mothers	906	906	0	0	41–66	12.6	2.81
Fathers	536	0	536	0	44–79	13.5	3.23
Total	3573	2143	1430	937	21–79	13.9	3.1

*EA* Educational Attainment (in years), *SD* Standard Deviation, *MZ* monozygotic, *DZ* dizygotic.

### Ethical approval

The TwinLife study received ethical approval from the German Psychological Association (protocol numbers: RR 11.2009 and RR 09.2013). Respondents provided written informed consent for their data to be used for research purposes^65^.

### Educational Attainment

Educational attainment was operationalised as a continuous variable by mapping the highest educational qualification obtained to a corresponding number of years of education (see Supplementary Table 6). Where twins or siblings were partway through a tertiary or professional qualification, we assigned years of education based on the completed qualification. In doing so we follow, Baier and Lang^60^, who note that German young adults who do not complete their enroled course generally achieve an alternative qualification of a similar type (e.g. tertiary or vocational) rather than dropping out. Means and standard deviations for the different types of family members are displayed in Table 6.

### Analyses

After calculating means and variances for each relative class, we calculated correlations between each type of family member (as shown in Table 3). We then corrected educational attainment for age and gender^66^ and z-standardised the residuals before fitting CTD or NTFD structural equation models. Twin modelling was performed using the OpenMx^67^ package in R^68^.

#### The Classical Twin Design (CTD)

The CTD is one of the most commonly used study designs in behavioural genetics. The CTD compares the resemblance of reared-together MZ twin for a given trait with the resemblance of reared-together DZ twins. The CTD assumes random mating on the trait in question, under which DZ twins are expected to share half of their trait relevant genes in common on average, compared to MZ twins who share all of their genes in common. The CTD also assumes that rearing conditions are equal between both kinds of twins (the Equal Environments Assumption), therefore any additional resemblance shown between MZ twin pairs compared to DZ twin pairs is attributed to additive genetic influence (*A*). Any residual similarity between MZ twins that is not explained by genetic influences is attributed to the shared environment (*C*). If MZ twins are more than twice as similar as DZ twins, genetic dominance is typically assumed to explain this, and it is modelled instead of *C*. Finally, the variance that cannot be accounted for by MZ twin resemblance is attributed to the nonshared environment (*E*). The methodology for fitting CTD structural equation models to twin data has been described in detail elsewhere^69^.

#### The Nuclear Twin and Family Design (NTFD)

Including additional relative classes in the NTFD enables several of the assumptions in the CTD to be relaxed and more parameters to be estimated. Non-additive genetic influences (*N*) and shared environmental influences can be estimated simultaneously, and the shared environment can be further decomposed into the shared sibling environment (*S*), the environmental effects of parental education on offspring education (*P*), and—if non-twin siblings are also included in the model—the twin-specific shared environment (*T*). Passive rGE can also be disaggregated from shared environmental influences. As means and variances in EA were similar for both twins and non-twin siblings (see Table 6), T was modelled as a variance component for all relative classes rather than an additional variance component experienced exclusively by twins^18,70^.

Incorporating data from parents also allows the NTFD model to directly account for assortative mating. We explored two boundary conditions: a phenotypic assortment model in which the correlation for EA between parents was assumed to be the result of active mate selection on education (inducing a genetic correlation between spouses), and a social homogamy model in which the correlation between spouses was assumed to be environmentally driven. We modelled social homogamy by extending the traditional NTFD model using innovations from the “Cascade” model developed by Keller et al. (2009)^20^. A latent phenotype (M’ and F’) is introduced between the observed parental phenotype (M and F) and the assortative mating copath (µ) linking each parent in the standard NTFD model.

Under the phenotypic assortment model, the variance of the latent parental phenotype is defined by the same variance components as the parental phenotype (and the variance of the parental phenotype is the same as its covariance with the latent phenotype making the algebra identical with that of the standard NTFD model). By contrast, under the social homogamy model, the genetic (*a* and *n*) paths leading to the latent phenotype are set to zero, obliging the covariance between the parental phenotype and the latent phenotype to be mediated by non-genetic factors.

As phenotypic transmission, non-additive genetic effects, and sibling-shared environmental influences could not all be estimated simultaneously^20^, we ran three baseline models (*ANSTE*, *AFSTE*, *ANFTE*) in which each of these three effects were respectively fixed to zero. This was performed under both a phenotypic assortment and a social homogamy assumption. These six baseline models were then compared against a saturated model (describing the means, variances and covariances of the different relative classes) using a chi-squared test to see if any produced a significantly worse fit to the data. For each baseline which did not show a significant reduction in fit, we iteratively dropped all paths with 95% confidence intervals crossing zero to see if this produced a significant reduction in fit using further chi-squared tests. Parameter estimates were reported for all baseline models which did not show a significant reduction in fit from the saturated model and were likewise reported for all submodels which did not show a significant reduction in fit compared to these baseline models. From these statistically significant models, the overall best-fitting model was determined on the basis of the lowest Akaike’s Information Criterion^71^. Finally, we ran a set of six additional baseline models to test if results were substantially affected if non-additive genetic effects were characterised as multi-local epistatic effects rather than as dominance or bi-local gene-gene interactions^22^.

**Fig. 3:**
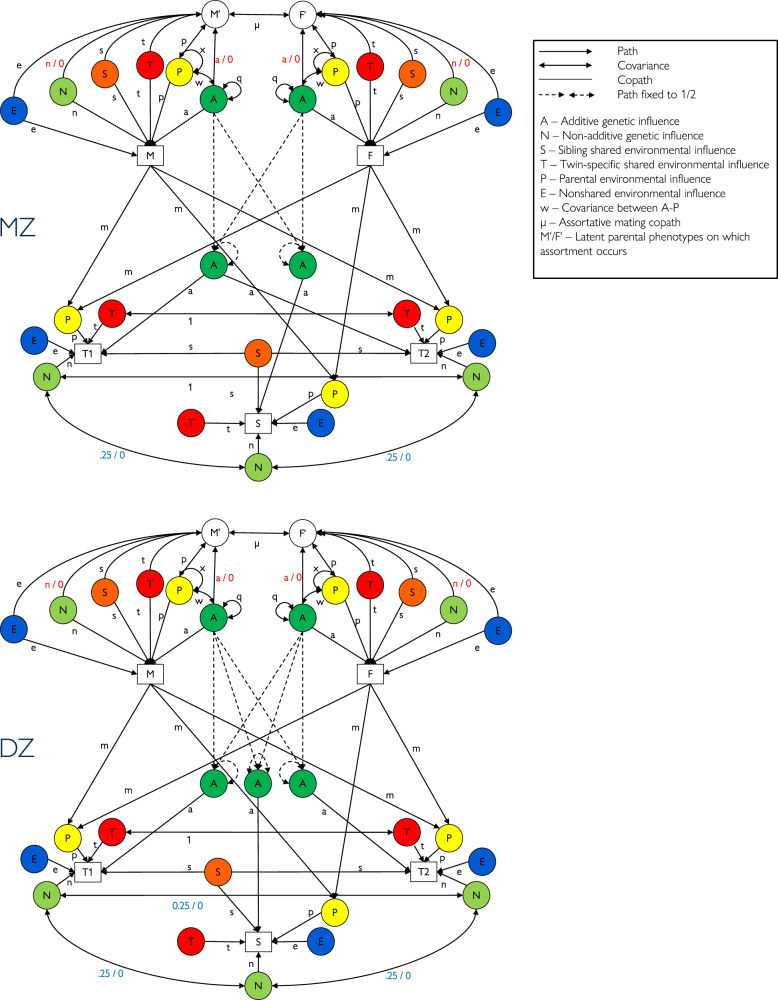
Path diagram of Nuclear Twin and Family Design (NTFD) structural equation model of educational attainment. The model for monozygotic (MZ) twins is displayed at the top and for dizygotic (DZ) twins at the bottom. Variances of latent factors are set to 1 unless otherwise specified (e.g., *q* = variance of *A* factor, *x* = variance of *P* factor). Path *p* was also set to 1 for model identification purposes. Social homogamy models set the genetic paths in red font to zero. A dominance model sets the non-additive genetic correlation between all siblings who are not MZ twins at 0.25 whereas an epistatic model sets that correlation at 0 (blue font).

A path diagram of our NTFD phenotypic assortment model is provided in Fig. 3. The algebra assumed to underlie our CTD, NTFD-PA, and NTFD-SH models is provided in Supplementary Table 7. The methodology for fitting NTFD structural equation models to twin and family data has been described in detail elsewhere^20^.

### Reporting summary

Further information on research design is available in the Nature Research Reporting Summary linked to this article.

## Supplementary information

Supplementary Notes

Supplementary Tables

Reporting Summary

## Data Availability

The TwinLife dataset that supports the main results of this study is available free of charge to researchers via GESIS (10.4232/1.13987) subject to the completion of a Data Use Agreement. The data used for adjusting grand mean ACE estimates in Branigan et al.^12^ for assortative mating are included in this published article (and its supplementary information files).

## Code availability

Code will be made available on request.

## References

[1] Geyer S, Hemström Ö, Peter R, Vågerö D (2006). Education, income, and occupational class cannot be used interchangeably in social epidemiology. Empirical evidence against a common practice. J. Epidemiol. Community Health.

[2] Lleras-Muney A (2005). The relationship between education and adult mortality in the United States. Rev. Econ. Stud..

[3] Lochner L, Moretti E (2004). The effect of education on crime: evidence from prison inmates, arrests, and self-reports. Am. Econ. Rev..

[4] Breen R, Jonsson JO (2005). Inequality of opportunity in comparative perspective: recent research on educational attainment and social mobility. Annu. Rev. Sociol..

[5] Hertz T (2008). The inheritance of educational inequality: international comparisons and fifty-year trends. BE J. Econ. Anal. Policy.

[6] van der Weide, R., Lakner, C., Mahler, D. G., Narayan, A. & Ramasubbaiah, R. *Intergenerational Mobility Around the World*. https://papers.ssrn.com/abstract=3981372. 10.2139/ssrn.3981372 (2021).

[7] Shavit, Y. & Blossfeld, H. *Persistent Inequality: Changing Educational Attainment In Thirteen Countries*. (Avalon Publishing, 1993).

[8] Grätz M (2021). Sibling similarity in education across and within societies. Demography.

[9] Jencks, C. & Tach, L. *Would Equal Opportunity Mean More Mobility?* https://papers.ssrn.com/abstract=779507. 10.2139/ssrn.779507 (2005).

[10] Taubman P (1976). The determinants of earnings: genetics, family, and other environments: a study of white male twins. Am. Econ. Rev..

[11] Taubman, P. *Kinometrics: Determinants of Socioeconomic Success Within and Between Families*. (North-Holland Publishing Company, 1977).

[12] Branigan AR, McCallum KJ, Freese J (2013). Variation in the heritability of educational attainment: an international meta-analysis. Soc. Forces.

[13] Silventoinen K (2020). Genetic and environmental variation in educational attainment: an individual-based analysis of 28 twin cohorts. Sci. Rep..

[14] Briley DA, Tucker-Drob EM (2017). Comparing the developmental genetics of cognition and personality over the Lifespan. J. Pers..

[15] Malanchini, M. et al. Pathfinder: A gamified measure to integrate general cognitive ability into the biological, medical and behavioural sciences. *bioRxiv* 2021.02.10.430571 10.1101/2021.02.10.430571 (2021). 10.1038/s41380-021-01300-0 PMC8872983 34599278

[16] Plomin R, Deary IJ (2015). Genetics and intelligence differences: five special findings. Mol. Psychiatry.

[17] Rimfeld K (2018). The stability of educational achievement across school years is largely explained by genetic factors. Npj Sci. Learn..

[18] Nielsen F, Roos JM (2015). Genetics of educational attainment and the persistence of privilege at the turn of the 21st Century. Soc. Forces.

[19] Freese J, Jao Y-H (2017). Shared environment estimates for educational attainment: a puzzle and possible solutions. J. Pers..

[20] Keller MC (2009). Modeling extended twin family data I: Description o. Twin Res. Hum. Genet..

[21] Keller MC, Medland SE, Duncan LE (2010). Are extended twin family designs worth the trouble? A comparison of the bias, precision, and accuracy of parameters estimated in four twin family models. Behav. Genet..

[22] Keller MC, Coventry WL (2005). Quantifying and Addressing Parameter Indeterminacy in the Classical Twin Design. Twin Res. Hum. Genet..

[23] Falconer, D. & MacKay, T. *Introduction to quantitative genetics*. (Longman, 1996). 10.1093/genetics/167.4.1529 PMC1471025 15342495

[24] Horwitz, T. B. & Keller, M. C. A comprehensive meta-analysis of human assortative mating in 22 complex traits. 2022.03.19.484997. Preprint at 10.1101/2022.03.19.484997 (2022).

[25] Heath AC, Eaves LJ (1985). Resolving the effects of phenotype and social background on mate selection. Behav. Genet..

[26] Zietsch BP, Verweij KJH, Heath AC, Martin NG (2011). Variation in human mate choice: simultaneously investigating heritability, parental influence, sexual imprinting, and assortative mating. Am. Nat..

[27] Yengo L (2018). Imprint of assortative mating on the human genome. Nat. Hum. Behav..

[28] Nivard, M. et al. Neither nature nor nurture: Using extended pedigree data to elucidate the origins of indirect genetic effects on offspring educational outcomes. Preprint at 10.31234/osf.io/bhpm5 (2022).

[29] Okbay A (2022). Polygenic prediction of educational attainment within and between families from genome-wide association analyses in 3 million individuals. Nat. Genet..

[30] Hugh-Jones D, Verweij KJH, St. Pourcain B, Abdellaoui A (2016). Assortative mating on educational attainment leads to genetic spousal resemblance for polygenic scores. Intelligence.

[31] Torvik FA (2022). Modeling assortative mating and genetic similarities between partners, siblings, and in-laws. Nat. Commun..

[32] Robinson MR (2017). Genetic evidence of assortative mating in humans. Nat. Hum. Behav..

[33] Martin, N. Genetics of sexual and social attitudes in twins. in *Twin Research: Psychology and Methodology, Alan R* 13–23 (1978). 568270

[34] Kendler KS, Ohlsson H, Lichtenstein P, Sundquist J, Sundquist K (2019). The Nature of the Shared Environment. Behav. Genet..

[35] Plomin R, DeFries JC (1980). Genetics and intelligence: Recent data. Intelligence.

[36] Lehti, H. *The role of kin in educational and status attainment*. (2020).

[37] Rietveld CA (2013). GWAS of 126,559 individuals identifies genetic variants associated with educational attainment. Science.

[38] Kong A (2018). The nature of nurture: Effects of parental genotypes. Science.

[39] Cheesman, R. et al. Comparison of Adopted and Nonadopted Individuals Reveals Gene–Environment Interplay for Education in the UK Biobank. *Psychol. Sci*. 0956797620904450 10.1177/0956797620904450 (2020). 10.1177/0956797620904450 PMC7238511 32302253

[40] Okbay A (2016). Genome-wide association study identifies 74 loci associated with educational attainment. Nature.

[41] Bates TC (2018). The nature of nurture: using a virtual-parent design to test parenting effects on children’s educational attainment in genotyped families. Twin Res. Hum. Genet..

[42] Eifler EF, Riemann R (2022). The aetiology of educational attainment: A nuclear twin family study into the genetic and environmental influences on school leaving certificates. Br. J. Educ. Psychol..

[43] Akaike, H. Factor Analysis and AIC. in *Selected Papers of Hirotugu Akaike* (eds. Parzen, E., Tanabe, K. & Kitagawa, G.) 371–386 (Springer New York, 1998). 10.1007/978-1-4612-1694-0_29.

[44] Raftery AE (1995). Bayesian model selection in social research. Sociol. Methodol..

[45] Lee JJ (2018). Gene discovery and polygenic prediction from a genome-wide association study of educational attainment in 1.1 million individuals. Nat. Genet..

[46] Young AI (2022). Discovering missing heritability in whole-genome sequencing data. Nat. Genet..

[47] Wainschtein P (2022). Assessing the contribution of rare variants to complex trait heritability from whole-genome sequence data. Nat Genet..

[48] Yengo L (2022). A saturated map of common genetic variants associated with human height. Nature.

[49] Engzell P, Tropf FC (2019). Heritability of education rises with intergenerational mobility. Proc. Natl Acad. Sci..

[50] Baier, T., Eilertsen, E. M., Ystrom, E., Zambrana, I. M. & Lyngstad, T. H. An Anatomy of the Intergenerational Correlation of Educational Attainment -Learning from the Educational Attainments of Norwegian Twins and their Children. *Res. Soc. Stratif. Mobil*. 100691 10.1016/j.rssm.2022.100691 (2022).

[51] Harden KP (2021). Reports of my death were greatly exaggerated”: behavior genetics in the postgenomic era. Annu. Rev. Psychol..

[52] Turkheimer E, D’Onofrio BM, Maes HH, Eaves LJ (2005). Analysis and interpretation of twin studies including measures of the shared environment. Child Dev..

[53] Engelhardt LE, Church JA, Harden KP, Tucker‐Drob EM (2019). Accounting for the shared environment in cognitive abilities and academic achievement with measured socioecological contexts. Dev. Sci..

[54] Rawls, J. *A Theory of Justice*. (Harvard University Press, 2009).

[55] Harden, K. P. *The Genetic Lottery: Why DNA Matters for Social Equality*. (Princeton University Press, 2021).

[56] deBoer, F. *The Cult of Smart: How Our Broken Education System Perpetuates Social Injustice*. (St. Martin’s Publishing Group, 2020).

[57] Hayek, F. A. von. *The Mirage of Social Justice*. (University of Chicago Press, 1978).

[58] Hayek, F. A. *The Constitution of Liberty: The Definitive Edition*. (Routledge, 2020).

[59] Morris, D. The Culture War is Coming for Your Genes. *Quillette* https://quillette.com/2021/09/30/the-culture-war-is-coming-for-your-genes/ (2021).

[60] Baier T, Lang V (2019). The social stratification of environmental and genetic influences on education: new evidence using a register-based twin sample. Sociol. Sci..

[61] Björklund, A. & Salvanes, K. G. Chapter 3 - Education and Family Background: Mechanisms and Policies. in *Handbook of the Economics of Education* (eds. Hanushek, E. A., Machin, S. & Woessmann, L.) vol. 3 201–247 (Elsevier, 2011).

[62] Holmlund H, Lindahl M, Plug E (2011). The causal effect of parents’ schooling on children’s schooling: a comparison of estimation methods. J. Econ. Lit..

[63] Young AI, Benonisdottir S, Przeworski M, Kong A (2019). Deconstructing the sources of genotype-phenotype associations in humans. Science.

[64] Lang, V. & Kottwitz, A. *The sampling design and socio-demographic structure of the first wave of the TwinLife panel study: a comparison with the Microcensus*. vol. 03 https://pub.uni-bielefeld.de/record/2913250 (2017).

[65] Lang V (2020). An introduction to the german twin family panel (TwinLife). Jahrb. F.ür. Natl Stat..

[66] McGue M, Bouchard TJ (1984). Adjustment of twin data for the effects of age and sex. Behav. Genet..

[67] Boker S (2011). OpenMx: An open source extended structural equation modeling framework. Psychometrika.

[68] R Core Team. *R: A language and environment for statistical computing*. (2020).

[69] Neale, M. & Cardon, L. R. *Methodology for Genetic Studies of Twins and Families*. (Springer Science & Business Media, 2013).

[70] Koeppen-Schomerus G, Spinath FM, Plomin R (2003). Twins and non-twin siblings: different estimates of shared environmental influence in early childhood. Twin Res. Hum. Genet..

[71] Wagenmakers E-J, Farrell S (2004). AIC model selection using Akaike weights. Psychon. Bull. Rev..

[72] Heath AC (1985). Education policy and the heritability of educational attainment. Nature.

[73] Lykken DT, Bouchard TJ, McGue M, Tellegen A (1990). The minnesota twin family registry: some initial findings. Acta Genet. Medicae Gemellol. Twin Res.

[74] Baker LA, Treloar SA, Reynolds CA, Heath AC, Martin NG (1996). Genetics of educational attainment in Australian twins: Sex differences and secular changes. Behav. Genet..

[75] Bingley, P., Christensen, K. & Walker, I. Twin-based Estimates of the Returns to Education: Evidence from the Population of Danish Twins. 30

[76] Silventoinen K, Sarlio-Lähteenkorva S, Koskenvuo M, Lahelma E, Kaprio J (2004). Effect of environmental and genetic factors on education-associated disparities in weight and weight gain: a study of Finnish adult twins. Am. J. Clin. Nutr..

[77] Silventoinen K, Kaprio J, Lahelma E (2000). Genetic and environmental contributions to the association between body height and educational attainment: a study of adult finnish twins. Behav. Genet..

[78] Ørstavik RE (2014). Sex differences in genetic and environmental influences on educational attainment and income. Twin Res. Hum. Genet..

[79] Lyngstad, T. H., Ystrøm, E. & Zambrana, I. M. An Anatomy of Intergenerational Transmission: Learning from the educational attainments of Norwegian twins and their parents. Preprint at 10.31235/osf.io/fby2t (2017).

[80] Isacsson G (1999). Estimates of the return to schooling in Sweden from a large sample of twins. Labour Econ..

