# Supplementary material for: Conventional twin studies overestimate the environmental differences between families relevant to educational attainment
Source: NPJ Sci Learn. 2023 Jul 17;8:24. doi: 10.1038/s41539-023-00173-y (PMC10352382; doi:10.1038/s41539-023-00173-y)
Supplement: Supplementary file 1 — Supplementary Notes [file 41539_2023_173_MOESM1_ESM.pdf]

Supplementary Notes for 'Conventional twin studies overestimate the environmental differences between families relevant to educational attainment'

Contents

Supplementary Notes for 'Conventional twin studies overestimate the environmental differences between families relevant to educational attainment' 1

Supplementary note 1: Estimates of genetic and environmental influence for educational attainment in the Polderman et al (2015) meta-analysis of twin studies 2

Supplementary note 2: Methods for our assortative mating adjustment to the estimates in the Branigan et al (2013) meta-analysis of twin studies of educational attainment 2

References 4

(N.B. Supplementary Tables are provided in a separate Excel file).

Supplementary note 1: Estimates of genetic and environmental influence for educational attainment in the Polderman et al (2015) meta-analysis of twin studies

In addition to the Branigan et al (2013)[1] meta-analysis and the Silventoinen et al (2020)[2] mega-analysis of international twin studies described in the main text, summary estimates of genetic and environmental influence for Educational Attainment (EA) are sometimes cited from a near exhaustive meta-analysis of ~50 years of twin studies by Polderman et al (2015)[3] (e.g. cited by Hugh-Jones et al., 2016[4] and Tropf et al., 2017[5]). The http://match.ctglab.nl/ interactive website released alongside the Polderman et al (2015) paper[3] reports a heritability of 52% and shared environmental influence of 27% for "education". However, this "education" phenotype is not restricted to years of education (including, e.g., some studies of school grades). Moreover, owing to the very large number of twin studies and phenotypes meta-analysed, the authors did not attempt to down-weight duplicate studies of the same samples, meaning some samples will have exerted an outsized influence on the meta-analytic results (see their supplementary Methods). For this reason, we focus on the results from the dedicated meta-analysis of EA by Branigan et al (2013)[1] and the dedicated mega-analysis by Silventoinen et al (2020).[2]

Supplementary note 2: Methods for our assortative mating adjustment to the estimates in the Branigan et al (2013) meta-analysis of twin studies of educational attainment

Estimates of additive genetic (*A*), shared environmental (*C*), and nonshared environmental influence (*E*) in the Classical Twin Design (CTD) can be biased due to the effects of assortative mating. We adapted algebra from Martin (1978)[6], and Baker et al (1996)[7] which adjusts CTD *ACE* estimates for assortative mating when data on parent or spousal correlations is available. We then identified thirteen of the thirty-four subgroups in the Branigan et al (2013)[1] meta-analysis of twin studies of EA which provided data on parental or spousal correlations (μ). For each of these subgroups, a parental genetic correlation (rG) was calculated using the formula $0.5(1-\sqrt{(1-4\mu A)})$. *C* was adjusted down using the formula $(A*rG)/(1-rG)$ until reaching zero. *A* was correspondingly adjusted up. This is equivalent to recalculating the Falconer's *ACE* estimates using a DZ twin kinship coefficient of 0.5 + 0.5rG rather than 0.5.

We proceeded to replicate the meta-analysis of *ACE* estimates that Branigan et al (2013)[1] had performed on the full sample on this subsample of studies with parent/spousal correlations. This required weighting each estimate by the inverse sampling variance (i.e., 1/(SE^2). To obtain the sampling variance for the Falconer's *ACE* estimates, we used the same formulas used by Branigan et al (2013).[1]

$$Var(A) = 4\left(\frac{(1-r\text{MZ}^2)^2}{n\text{MZ}-1} + \frac{(1-r\text{DZ}^2)^2}{n\text{DZ}-1}\right) \qquad (1)$$

$$Var(C) = 4\left(\frac{(1-r\text{DZ}^2)^2}{n\text{DZ}-1} + \frac{(1-r\text{MZ}^2)^2}{n\text{MZ}-1}\right) \qquad (2)$$

$$Var(E) = \frac{(1-r\text{MZ}^2)^2}{n\text{MZ}-1} \qquad (3)$$

After calculating the grand mean for the unadjusted *ACE* estimates in this subsample, we then used the same weights to calculate the grand mean for the *adjusted ACE* estimates after these subgroups had been individually corrected for assortative mating. The adjustment for the mean heritability estimate (18%) was slightly different from the adjustment for the mean shared environmental estimate (17%) because the weights for each variance component were calculated separately.

A previous study by Kemper et al (2021)[9] calculated assortative mating adjusted variance components for Branigan et al (2013)[1] of *A* = 49% and *C* = 25% (from *A* = 40%, *C* = 36%). This re-allocation of 9% of the *C* variance was calculated using similar algebra to that we employ to adjust each individual study. However, whereas we apply an adjustment to each study based on parent/spousal correlations reported in that study and then meta-analyse the results, Kemper et al. correct the grand mean ACE estimates from Branigan et al (2013)[1] assuming a single overall spousal correlation of 0.42 applies to the whole sample. One issue here is that the 0.42 spousal correlation these authors employ was calculated from studies that were not in the Branigan et al[1] meta-analysis and might not be relevant for these samples. Kemper et al[9] calculated this value by meta-analysing spousal correlations from the UK Biobank and the US sample in Price and Vandenberg (1980)[10], with only one study from Branigan et al[1] included — an Australian study by Baker et al (1996)[7]. In Supplementary Table 8 we replicate Kemper et al's meta-analysis using

parent/spousal correlations from studies included in Branigan et al[1]. This produces a substantially higher mean spousal correlation of 0.57. If we adjust the Branigan et al (2013)[1] *ACE* estimate applying Kemper et al's algebra to this revised value, 22 percentage points of the *C* variance are re-allocated to the *A* component, implying adjusted estimates of *A* = 62% and *C* = 14%.

The main cause of the discrepancy between our (preferred) main estimate based on the weighted average adjustment to *each* subgroup vs. the average spousal correlation across *all* studies is due to the size of the adjustment being bounded in the first instance by the *C* estimate in each subgroup. This limits the effect of large studies with high spousal correlations on the overall adjustment. Nevertheless, this bounded estimate still suggests an adjustment nearly twice as large as that calculated in Kemper et al (2021), i.e., 16-17% rather than 9%.

References

1. Branigan, A. R., McCallum, K. J. & Freese, J. Variation in the Heritability of Educational Attainment: An International Meta-Analysis. *Soc. Forces* **92**, 109–140 (2013).
2. Silventoinen, K. *et al.* Genetic and environmental variation in educational attainment: an individual-based analysis of 28 twin cohorts. *Sci. Rep.* **10**, 12681 (2020).
3. Polderman, T. J. C. *et al.* Meta-analysis of the heritability of human traits based on fifty years of twin studies. *Nat. Genet.* **47**, 702–709 (2015).
4. Hugh-Jones, D., Verweij, K. J. H., St. Pourcain, B. & Abdellaoui, A. Assortative mating on educational attainment leads to genetic spousal resemblance for polygenic scores. *Intelligence* **59**, 103–108 (2016).
5. Tropf, F. C. *et al.* Hidden heritability due to heterogeneity across seven populations. *Nat. Hum. Behav.* **1**, 757–765 (2017).
6. Martin, N. Genetics of sexual and social attitudes in twins. in *In Twin Research: Psychology and Methodology, Alan R* 13–23 (1978).

7. Baker, L. A., Treloar, S. A., Reynolds, C. A., Heath, A. C. & Martin, N. G. Genetics of educational attainment in Australian twins: Sex differences and secular changes. *Behav. Genet.* **26**, 89–102 (1996).
8. Nielsen, F. & Roos, J. M. Genetics of Educational Attainment and the Persistence of Privilege at the Turn of the 21st Century. *Soc. Forces* **94**, 535–561 (2015).
9. Kemper, K. E. *et al.* Phenotypic covariance across the entire spectrum of relatedness for 86 billion pairs of individuals. *Nat. Commun.* **12**, 1050 (2021).
10. Price, R. A. & Vandenberg, S. G. Spouse similarity in American and Swedish couples. *Behav. Genet.* **10**, 59–71 (1980).
11. Falconer, D. & MacKay, T. *Introduction to quantitative genetics*. (Longman, 1996).
12. Kong, A. *et al.* The nature of nurture: Effects of parental genotypes. *Science* **359**, 424–428 (2018).
13. Young, A. I., Benonisdottir, S., Przeworski, M. & Kong, A. Deconstructing the sources of genotype-phenotype associations in humans. *Science* **365**, 1396–1400 (2019).
