# Supplementary material for: Conventional twin studies overestimate the environmental differences between families relevant to educational attainment
Source: NPJ Sci Learn. 2023 Jul 17;8:24. doi: 10.1038/s41539-023-00173-y (PMC10352382; doi:10.1038/s41539-023-00173-y)
Supplement: Supplementary file 3 — Reporting Summary [file 41539_2023_173_MOESM3_ESM.pdf]

nature portfolio

Corresponding author(s): Tobias Wolfram

Last updated by author(s): Feb 17, 2023

Reporting Summary

Nature Portfolio wishes to improve the reproducibility of the work that we publish. This form provides structure for consistency and transparency in reporting. For further information on Nature Portfolio policies, see our Editorial Policies and the Editorial Policy Checklist.

Statistics

For all statistical analyses, confirm that the following items are present in the figure legend, table legend, main text, or Methods section.

n/a	Confirmed	
☐	☒	The exact sample size (*n*) for each experimental group/condition, given as a discrete number and unit of measurement
☐	☒	A statement on whether measurements were taken from distinct samples or whether the same sample was measured repeatedly
☐	☒	The statistical test(s) used AND whether they are one- or two-sided<br>*Only common tests should be described solely by name; describe more complex techniques in the Methods section.*
☐	☒	A description of all covariates tested
☐	☒	A description of any assumptions or corrections, such as tests of normality and adjustment for multiple comparisons
☐	☒	A full description of the statistical parameters including central tendency (e.g. means) or other basic estimates (e.g. regression coefficient) AND variation (e.g. standard deviation) or associated estimates of uncertainty (e.g. confidence intervals)
☐	☒	For null hypothesis testing, the test statistic (e.g. *F*, *t*, *r*) with confidence intervals, effect sizes, degrees of freedom and *P* value noted<br>*Give P values as exact values whenever suitable.*
☒	☐	For Bayesian analysis, information on the choice of priors and Markov chain Monte Carlo settings
☒	☐	For hierarchical and complex designs, identification of the appropriate level for tests and full reporting of outcomes
☐	☒	Estimates of effect sizes (e.g. Cohen's *d*, Pearson's *r*), indicating how they were calculated

Our web collection on statistics for biologists contains articles on many of the points above.

Software and code

Policy information about availability of computer code

Data collection	Does not apply (secondary data analysis)
Data analysis	R

For manuscripts utilizing custom algorithms or software that are central to the research but not yet described in published literature, software must be made available to editors and reviewers. We strongly encourage code deposition in a community repository (e.g. GitHub). See the Nature Portfolio guidelines for submitting code & software for further information.

Data

Policy information about availability of data

All manuscripts must include a data availability statement. This statement should provide the following information, where applicable:

- Accession codes, unique identifiers, or web links for publicly available datasets
- A description of any restrictions on data availability
- For clinical datasets or third party data, please ensure that the statement adheres to our policy

A data availability statement has been added to the text.

Human research participants

Policy information about studies involving human research participants and Sex and Gender in Research.

Reporting on sex and gender	No differential sex/gender information was available.
Population characteristics	German twins in early adulthood and their parents
Recruitment	Secondary analysis of a probability sample of the german twin population (TwinLife).
Ethics oversight	The TwinLife study received ethical approval from the German Psychological Association (protocol numbers: RR 11.2009 and RR 09.2013)

Note that full information on the approval of the study protocol must also be provided in the manuscript.

Field-specific reporting

Please select the one below that is the best fit for your research. If you are not sure, read the appropriate sections before making your selection.

☐ Life sciences ☒ Behavioural & social sciences ☐ Ecological, evolutionary & environmental sciences

For a reference copy of the document with all sections, see nature.com/documents/nr-reporting-summary-flat.pdf

Behavioural & social sciences study design

All studies must disclose on these points even when the disclosure is negative.

Study description	Quantitative study (nuclear twin family design)
Research sample	The German Twin Family Panel (TwinLife) is a German longitudinal study of monozygotic and dizygotic same-sex twin pairs and their families that was designed to investigate the development of social inequalities over the life course. The study covers an observation period from approximately 2014 to 2023. The target population of the sample are reared-together twins of four different age cohorts that were born in 2009/ 2010 (cohort 1), in 2003/2004 (cohort 2), in 1997/1998 (cohort 3) and between 1990 and 1993 (cohort 4). In the first wave, the study included data on 4097 twin families. Families were recruited in all parts of Germany so that the sample comprises the whole range of the educational, occupational and income structure. For this study, we used twin families from cohort 4.
Sampling strategy	TwinLife is the first twin (family) panel in Germany implementing a population register-based sampling design. Details are given in https://www.twin-life.de/documentation/images/TwinLife/Downloads/twinlife_tr_05.pdf
Data collection	Data was collected in a combination of computer assisted telephone and face to face interviews, see https://www.twin-life.de/documentation/images/TwinLife/Downloads/twinlife_tr_05.pdf for details.
Timing	Data comprises the first two twinlife waves and was collected from 2014-2017.
Data exclusions	No cases were excluded.
Non-participation	The response rate for wave 1 of twinlife was 36% (https://www.twin-life.de/documentation/images/TwinLife/Downloads/twinlife_tr_05.pdf)
Randomization	No experimental design was used

Reporting for specific materials, systems and methods

We require information from authors about some types of materials, experimental systems and methods used in many studies. Here, indicate whether each material, system or method listed is relevant to your study. If you are not sure if a list item applies to your research, read the appropriate section before selecting a response.

Materials & experimental systems

n/a	Involved in the study
☒	☐ Antibodies
☒	☐ Eukaryotic cell lines
☒	☐ Palaeontology and archaeology
☒	☐ Animals and other organisms
☒	☐ Clinical data
☒	☐ Dual use research of concern

Methods

n/a	Involved in the study
☒	☐ ChIP-seq
☒	☐ Flow cytometry
☒	☐ MRI-based neuroimaging
